# The complete mitochondrial genome of *Tephrinectes sinensis* and its phylogenetic position relative to Paralichthyidae

**DOI:** 10.1080/23802359.2025.2569567

**Published:** 2025-10-07

**Authors:** Wu-Wei Chen, Yu-Hui Tao, Yin-Tao Zhang, Jin-Qiang Cheng, Jin-Yang Li, Cheng-Wei Tong, Jie Chen, Wei Liu

**Affiliations:** ^a^ Zhejiang Lishui Service Platform for Technological Innovations in Traditional Chinese Medicine Industry (ZJLS-TCMTI SP), Lishui University, Lishui, China; ^b^ Qingyuan Bureau of Natural Resources and Planning, Lishui, China; ^c^ Forestry Bureau of Jinyun County, Lishui, China; ^d^ College of Ecology, Lishui University, Lishui, China; ^e^ Forestry Bureau of Lishui City, Lishui, China

**Keywords:** Mitogenome assembly, phylogenetic analysis, flatfish evolution

## Abstract

*Tephrinectes sinensis*, an endemic flatfish from China’s coastal waters, has remained taxonomically contentious owing to its distinctive morphology and osteology. We sequenced and annotated the first complete mitochondrial genome of this species using Illumina HiSeq 2500. The circular mitogenome spans 17,366 bp, encoding 13 protein-coding genes (PCGs), 22 tRNA genes, two rRNA genes, and a control region. A maximum-likelihood phylogeny places *T. sinensis* as a single-taxon lineage sister to the remaining Paralichthyidae. This matrilineal pattern is congruent with the long-standing morphological debate over its familial placement.The complete mitogenome provides a foundational genomic resource for resolving relationships within Pleuronectiformes.

## Introduction

*Tephrinectes sinensis* (Lacepède, 1802), a warm-temperate demersal fish endemic to coastal China (Pleuronectiformes: Paralichthyidae), exhibits distinctive morphological features: marked bilateral vertebral asymmetry, a laterally compressed ovoid body with a broad caudal peduncle, and left-sided ocular placement (Hoshino and Amaoka 1998). The ocular side displays a brownish background with melanic speckles and yellowish fins bearing dark markings, contrasting with the blind side’s uniform white coloration (Chapleau 1993). In aquaculture conditions, specimens primarily consume soft prey such as fish and shrimp flesh.

Notably, Hoshino and Amaoka (1998) challenged conventional classification through comparative hypural morphology and anatomical analyses, proposing reclassification from Paralichthyidae to the suborder Pleuronectoidei. This hypothesis aligns with Hensley’s earlier phylogenetic framework (Hensley et al. 1984), though taxonomic consensus remains unresolved.

This work reports the first complete mitochondrial genome of *T. sinensis*, providing a high-resolution matrilineal marker to assess its phylogenetic placement relative to Paralichthyidae and a genomic foundation for subsequent multilocus phylogenomic studies within Pleuronectiformes.

## Materials and methods

**Figure 1.**
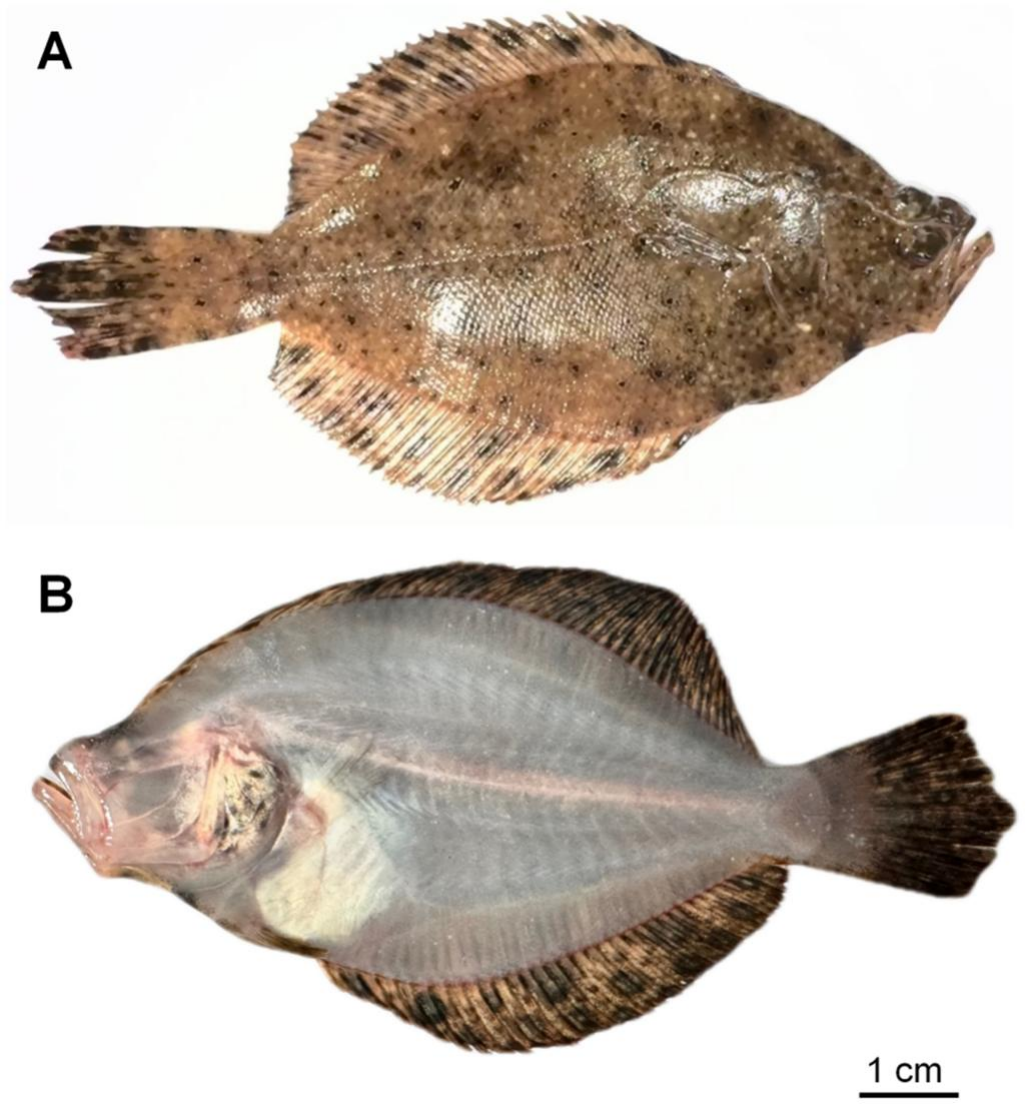
Reference images of *Tephrinectes sinensis*. (A) Ocular side (dorsal view). (B) Blind side (ventral view). Both photographs were taken by the author of this article, Wu-Wei Chen.

In December 2024, field sampling of *T. sinensis* was conducted in Shanwei City, China (22°47′14″N, 115°22′32″E), where species-level identification was achieved using established taxonomic keys following the protocol (Hoshino and Amaoka 1998). Notable identification features of *T. sinensis* include a tilted second dorsal spine that covers the skull and left-sided ocular placement. Subsequent to collection, digital imaging of specimens was performed using a Nikon D850 digital SLR system (Minato City, Japan) prior to humane euthanasia through eugenol overdose administration. Post-procedural tissue sampling involved the excision of muscle tissue from imaged specimens, which were immediately stored in 95% ethanol for molecular preservation. The remaining specimens underwent complete fixation in 95% ethanol prior to archival deposition at the College of Ecology’s zoological repository (Lishui University). These accessioned specimens have been formally assigned the catalog number LSU-2024-12-0125 (Figure 1), with Dr. Jie Chen (jchen@lsu.edu.cn) designated as the contact person.

Genomic DNA isolation was performed on muscle samples employing the Rapid Animal Genomic DNA Isolation Kit (Sangon Biotech, Shanghai, China), following manufacturer’s protocols. DNA libraries with a 350-bp insert size were constructed using the TruSeq Nano™ kit (Illumina, San Diego, CA) and sequenced on the Illumina HiSeq 2500 platform, generating 150-bp paired-end reads. Initial sequencing output reached 10.90 Gb, with 10.28 Gb retained after quality filtration processes. Quality control and adapter trimming were performed using Fastp v0.20.0 (Chen et al. 2018), yielding 77,061,072 filtered paired-end reads that were used for mitochondrial genome reconstruction. Quality-filtered reads (77,061,272 paired-end sequences) were first mapped to the *Paralichthys olivaceus* reference mitogenome (GenBank: AB028664; Saitoh et al. 2000) using BWA-MEM v0.7.17 (Li 2013). Mapped reads were assembled with SPAdes v4.10 (Prjibelski et al. 2020; k-mer: 21,33,55), generating a 17,366 bp draft contig. Crucially, the identical read set was independently subjected to *de novo* assembly using GetOrganelle v1.7.7 (Jin et al. 2020) with extended k-mer ranges (21–127), yielding an identical 17,366 bp contig that contained all 37 canonical mitochondrial elements. This congruence confirmed absence of reference bias and sample contamination. The consensus contig was circularized using MitoZ v2.4 (Meng et al. 2019), polished twice with Pilon v1.24 (Walker et al. 2014) for error correction, and annotated through a multi-validation pipeline: structural predictions from MITOS2 (Donath et al. 2019) and MiTFi (Jühling et al. 2012) were cross-verified via homology searches (NCBI BLAST+ v2.28; Camacho et al. 2009), gene prediction (GeneWise; Birney et al. 2004), and structural RNA detection (Infernal v1.1; Nawrocki and Eddy 2013). All annotations were manually curated in Geneious Prime v2024.0.7 (Geneious 2025). Coverage depth was validated by realigning raw reads to the final assembly (Bowtie2 v2.3.4; Langmead and Salzberg 2012), with per-base depth calculated via SAMtools v1.16.1 (Li et al. 2009) and visualized using ggplot2 (Wickham 2016; Fig. S1).

For phylogenetic reconstruction, we retrieved the complete mitochondrial genomes available for the family Paralichthyidae (14 species), along with the novel *T. sinensis* sequence acquired in this investigation. The complete mitogenomes of *Takifugu rubripes* and *Lateolabrax japonicus* served as outgroup taxa in our phylogenetic analyses. Initial data processing involved systematic extraction of 13 protein-coding genes (PCGs) through PhyloSuite v1.2.1 (Zhang et al. 2020), followed by comparative sequence alignment using MAFFT v7.388’s alignment algorithms (Katoh and Standley 2013). Molecular phylogenetic inference was performed through maximum-likelihood methodology in IQ-TREE v2.1.2 (Minh et al. 2020), implementing the optimal GTR + F + I + G4 substitution model identified via ModelFinder’s statistical evaluation (Kalyaanamoorthy et al. 2017). Topological robustness was assessed through 1000 bootstrap resampling iterations. Final phylogenetic visualization and metadata annotation were executed using the Interactive Tree of Life platform (ITOL v6) (Letunic and Bork 2021).

## Results

**Figure 2.**
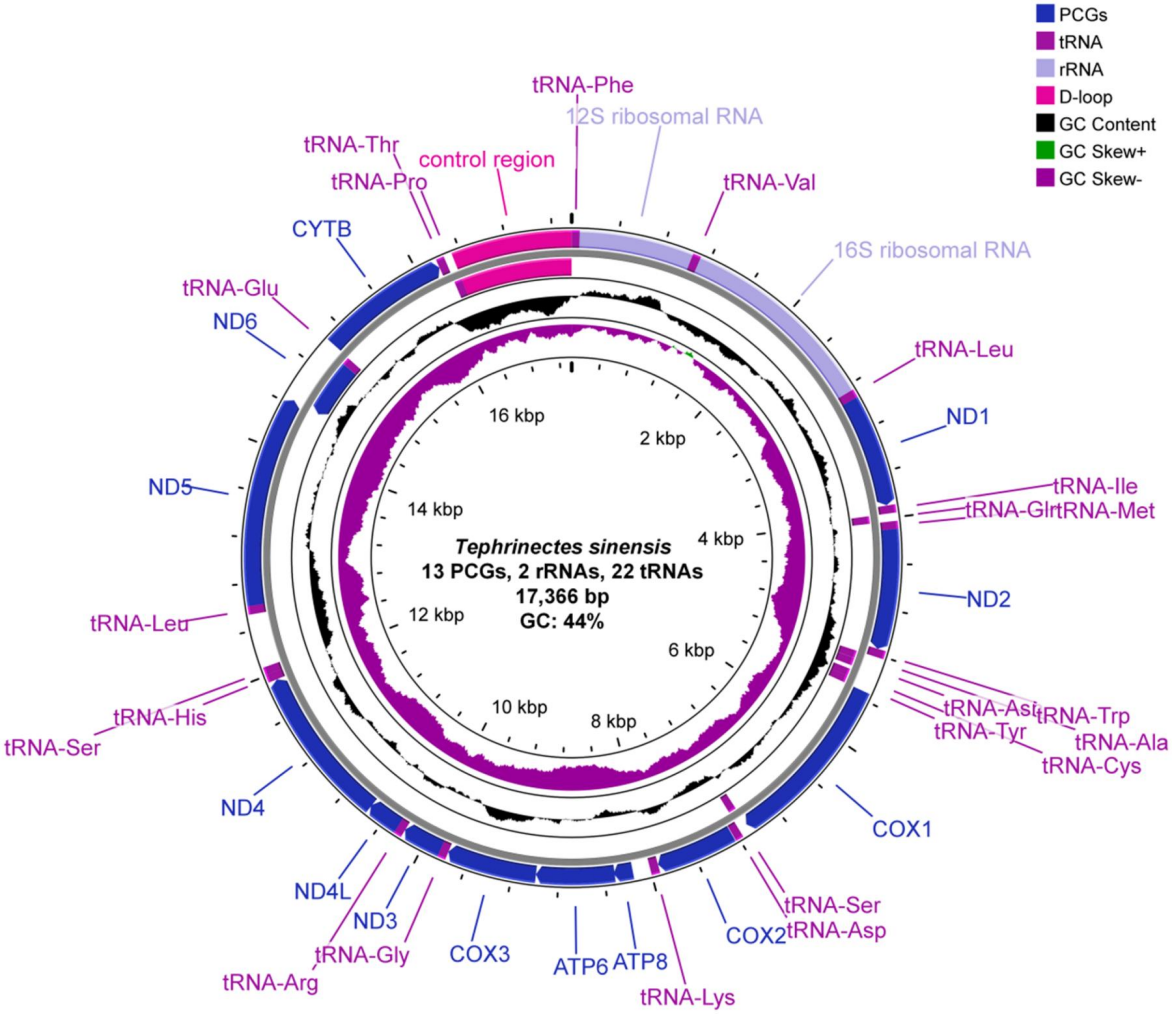
Circular map of the mitochondrial genome of *T. sinensis*. From inner to outer rings: GC-skew, GC content, and gene distribution. GC content was computed per sliding window (500-bp window; 1-bp step) and plotted as deviation (%) from the genome-wide mean (baseline at 0%); GC-skew was computed as (G − C)/(G + C) and is shown on an absolute scale. Genes are depicted on their transcribed strand, with arrows indicating transcriptional direction; the non-coding control region is shown without strand designation. Gene types and GC content/skew are color-coded. Labels for protein-coding genes denote their protein products (roman type) rather than italicized gene symbols; tRNAs are labeled by amino-acid identity; rRNA genes are labeled by their RNA products (roman) rather than italicized gene symbols.

The mitochondrial architecture of *T. sinensis* exhibited characteristic conservation, presenting a circular 17,366 bp genome organized into 37 functional elements: 13 PCGs, 22 transfer RNA genes, two ribosomal RNA genes, and a non-coding regulatory domain. Nucleotide distribution analysis revealed base composition asymmetry (A: 28.61%, T: 27.28%, G: 16.67%, and C: 27.44%) with an aggregate GC content of 44%. Strand-specific gene distribution showed H-strand encoding 28 elements (12 PCGs, 14 tRNA genes, and rRNA clusters), while L-strand harbored nine genetic elements (*ND6* PCG + eight tRNA genes). Using the vertebrate mitochondrial code, most PCGs start with ATG, whereas *COX1*, *ATP6*, and *ND3* start with GTG. Termination patterns comprised complete (TAA/TAG) and incomplete stop codons, the latter observed in *COX2*, *ND4*, and *CYTB* genes requiring polyadenylation for functional completion. tRNA gene lengths ranged from 67 to 75 bp, whereas ribosomal RNA genes showed length differences (*rrnL* = 1730 bp; *rrnS* = 982 bp) and distinct GC profiles (42.83% vs. 47.76%). A 1037-bp non-coding region – putatively corresponding to the displacement loop (D-loop) – was detected between *trnF* (tRNA-Phe) and *trnP* (tRNA-Pro); its GC content is 36.16% (Figure 2).

**Figure 3.**
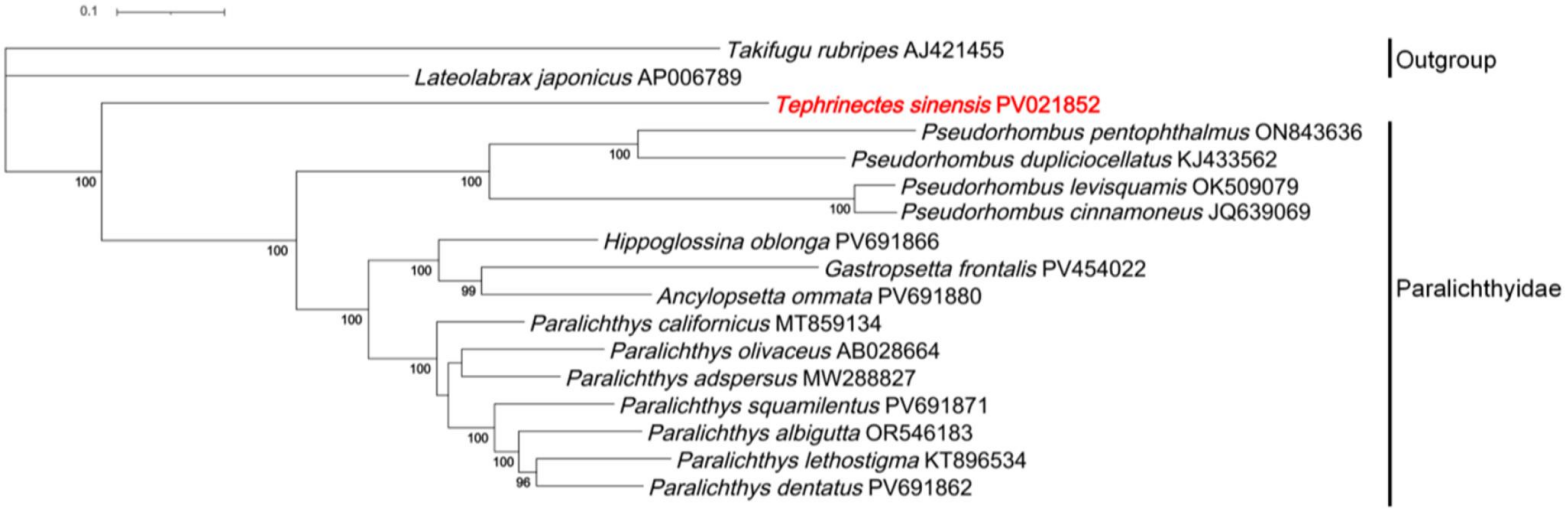
Maximum-likelihood phylogenetic tree based on the whole mitogenomes of *T. sinensis* and 14 species of Paralichthyidae family. Bootstrap values >70% are displayed above the branches. The following sequences were used: *Tephrinectes sinensis* (PV021852; this study), *Paralichthys olivaceus* (AB028664; Saitoh et al. 2000), *Paralichthys adspersus* (MW288827; Marín et al. 2021), *Paralichthys californicus* (MT859134; Vargas-Peralta et al. 2020), *Paralichthys squamilentus* (PV691871; unpublished), *Paralichthys albigutta* (OR546183; unpublished), *Paralichthys lethostigma* (KT896534; Shi et al. 2016), *Paralichthys dentatus* (PV691862; Xu et al. 2016), *Gastropsetta frontalis* (OR546157; unpublished), *Ancylopsetta ommata* (OR546182; unpublished), *Hippoglossina oblonga* (OP056997; unpublished), *Pseudorhombus dupliciocellatus* (KJ433562; Si et al. 2017), *Pseudorhombus pentophthalmus* (ON843636; unpublished), *Pseudorhombus levisquamis* (OK509079; unpublished), *Pseudorhombus cinnamoneus* (JQ639069; Shi et al. 2013), *Takifugu rubripes* (AJ421455; Elmerot et al. 2002), *Lateolabrax japonicus* (AP006789; Lavoué et al. 2014).

Under the *a priori* outgroup rooting (*Lateolabrax japonicus*, *Takifugu rubripes*), the maximum-likelihood tree (Figure 3) recovers a monophyletic Paralichthyidae with generally high node support. Notably, *T. sinensis* forms a single-taxon lineage that is sister to all remaining paralichthyid taxa rather than nesting within any sampled genus. Within Paralichthyidae, branching depths are heterogeneous: some clades (e.g. the Paralichthys cluster) exhibit short internodes indicative of shallow divergences, whereas deeper splits separate other genera. Together, these features constitute a complex differentiation pattern – the coexistence of an early-diverging single lineage and a set of uneven, hierarchically structured divergences – rather than a simple, uniform split among taxa.

## Discussion and conclusions

Here, we report the complete mitochondrial genome of *T. sinensis*. Under the vertebrate mitochondrial code (translation table 2), most PCGs initiate with ATG, whereas *COX1*, *ATP6*, and *ND3* initiate with GTG – a pattern also reported in teleosts such as *Gobio huanghensis* (Du et al. 2024), *Protonibea diacanthus* (Liu et al. 2016), and *Rasbora tornieri* (Chung et al. 2020). The control region is 1037 bp long with a GC content of 36.16%. This relatively long, AT-rich control region may represent lineage-specific variation in non-coding mitochondrial DNA and could serve as a useful marker for future population-level studies.

Phylogenetic reconstruction based on 14 paralichthyid mitogenomes, under an *a priori* outgroup rooting, recovered with maximal support (bootstrap = 100%) that *T. sinensis* forms a single, early-diverging lineage sister to the remaining Paralichthyidae, rather than nesting within any sampled genus. This matrilineal signal provides molecular corroboration for the morphological hypothesis advocating its exclusion from Paralichthyidae (Hoshino and Amaoka 1998), although nuclear-genome analyses are still required to determine whether it reflects species-level divergence or mito-nuclear discordance. The topology also reveals a complex differentiation pattern within the family – heterogeneous branch lengths and uneven divergence depths with short-internode clusters – consistent with morphology-based syntheses that portray bothid relationships as mosaic and hierarchically non-uniform (Hensley et al. 1984; Chapleau 1993). Notably, despite deep splits, the conserved mitogenomic architecture parallels models of incremental lateralization (Frazzetta 2012), hinting at coordination between genomic stability and key flatfish innovations (e.g. ocular migration, vertebral asymmetry). Figure 3 visualizes these expectations while clarifying the distinct placement of *T. sinensis*.

The complete mitogenome of *T. sinensis* makes two contributions to flatfish systematics: (1) under an *a priori* outgroup-rooted mtDNA analysis, it places *T. sinensis* as a single-taxon lineage sister to, rather than nested within, Paralichthyidae; and (2) it provides a high-fidelity matrilineal reference for comparative work across Pleuronectiformes. Because mitochondrial genomes constitute a single, maternally inherited, nonrecombining locus, these inferences are provisional; integrated multilocus nuclear analyses are required for definitive taxonomic resolution.

## Supplementary Material

Figure S1.doc

## Data Availability

The genome sequence data supporting this study are openly available in GenBank of NCBI at https://www.ncbi.nlm.nih.gov under the accession number PV021852. The associated BioProject, SRA, and Biosample numbers are PRJNA1215796, SRR32129885, and SAMN46419712, respectively.
