# Supplementary material for: The complete mitochondrial genome of Tephrinectes sinensis and its phylogenetic position relative to Paralichthyidae
Source: Mitochondrial DNA B Resour. 2025 Oct 7;10(11):1021–5. doi: 10.1080/23802359.2025.2569567 (PMC12507108; doi:10.1080/23802359.2025.2569567)
Supplement: Figure S1.doc [file TMDN_A_2569567_SM5047.doc]

**The complete mitochondrial genome of *Tephrinectes sinensis* (Pleuronectiformes) and its implications for taxonomic reclassification within Pleuronectoidei**

Wu-Wei Chen, Yu-Hui Tao, Yin-Tao Zhang, Jin-Qiang Cheng, Jin-Yang Li, Cheng-Wei Tong, Jie Chen, Wei Liu

**Figure S1.** Depth of coverage for *Tephrinectes sinensis* mitochondrial genome. X and Y axis present nucleotide position of *T. sinensis* mitochondrial genome and coverage depth, respectively.
